# Balancing scarce hospital resources during the COVID-19 pandemic using discrete-event simulation

**DOI:** 10.1007/s10729-021-09548-2

**Published:** 2021-04-09

**Authors:** G.J. Melman, A.K. Parlikad, E.A.B. Cameron

**Affiliations:** 1 Institute for Manufacturing, Department of Engineering, University of Cambridge, 17 Charles Babbage Rd, Cambridge, CB3 0FS UK; 2 Modelling Support, Addenbrooke’s Hospital, Cambridge University Hospitals NHS Foundation Trust, Cambridge, UK; 3 Industrial Engineering & Innovation Sciences, Eindhoven University of Technology, Eindhoven, Netherlands; 4 Consultant Gastroenterologist and Director of Improvement and Transformation, Addenbrooke’s Hospital, Cambridge University Hospitals NHS Foundation Trust, Box 146 Executive Offices, Cambridge, CB2 0QQ UK

**Keywords:** Resource allocation, COVID-19, Simulation, Capacity management, Intensive care, Operations research

## Abstract

COVID-19 has disrupted healthcare operations and resulted in large-scale cancellations of elective surgery. Hospitals throughout the world made life-altering resource allocation decisions and prioritised the care of COVID-19 patients. Without effective models to evaluate resource allocation strategies encompassing COVID-19 and non-COVID-19 care, hospitals face the risk of making sub-optimal local resource allocation decisions. A discrete-event-simulation model is proposed in this paper to describe COVID-19, elective surgery, and emergency surgery patient flows. COVID-19-specific patient flows and a surgical patient flow network were constructed based on data of 475 COVID-19 patients and 28,831 non-COVID-19 patients in Addenbrooke’s hospital in the UK. The model enabled the evaluation of three resource allocation strategies, for two COVID-19 wave scenarios: proactive cancellation of elective surgery, reactive cancellation of elective surgery, and ring-fencing operating theatre capacity. The results suggest that a ring-fencing strategy outperforms the other strategies, regardless of the COVID-19 scenario, in terms of total direct deaths and the number of surgeries performed. However, this does come at the cost of 50% more critical care rejections. In terms of aggregate hospital performance, a reactive cancellation strategy prioritising COVID-19 is no longer favourable if more than 7.3% of elective surgeries can be considered life-saving. Additionally, the model demonstrates the impact of timely hospital preparation and staff availability, on the ability to treat patients during a pandemic. The model can aid hospitals worldwide during pandemics and disasters, to evaluate their resource allocation strategies and identify the effect of redefining the prioritisation of patients.

## Highlights

- Bridges the gap between pandemic and non-pandemic capacity management models by holistically evaluating both patient groups
- Provides an open-source and modifiable simulation model to capture COVID-19 and non-COVID-19 patient flows
- Enables hospitals to visualise and quantify effect of resource allocation and patient prioritisation decisions
- Demonstrates the importance for hospitals to proactively train the surgical workforce to work on critical care

## Introduction

Across the globe, healthcare systems have been rapidly transformed by the COVID-19 (SARS-CoV-2) pandemic. The virus has impacted people directly infected by the virus, and those indirectly affected by the consequences of the virus. Healthcare systems are under immense pressure, and governments introduced public health measures to ’suppress’ the wave of infections and alleviate the subsequent pressure on hospitals. In order to prepare for and respond to this disaster, hospitals engaged in two efforts to cope with this influx, by i) predicting COVID-19 hospital admissions and resource requirements, and ii) building the capacity to treat COVID-19 patients optimally. This response suggests that the focus is solely on COVID-19 patients. However, the need for non-COVID-19 care did not disappear, and patients still need their urgently required healthcare. For the United Kingdom (UK) alone, it is expected that 1-3 million people will be awaiting surgery by 2021 [20]. Therefore, it is essential from an ethical and strategic point-of-view to focus on both COVID-19 and non-COVID-19 care by allocating the scarce resources to both patient groups. This paper uses a discrete-event simulation model to evaluate the impact of hospital resource allocation decisions on both patient groups. This study was carried out as part of hospital’s operational response to the pandemic. We now provide a brief review of the literature on predicting resources during a pandemic and allocating scarce resources.

### Pandemic resource prediction modelling

COVID-19 places a substantial burden on a range of resources, including oxygen, general ward (GW) beds, intensive care (ICU) beds, ventilators, anaesthetists, intensivists, nursing staff (RN), mortuary, consumables, and personal protection equipment (PPE). Combined with the push over the last decades to increase resource utilisation and minimise safety stock levels through just-in-time principles, hospitals now face extreme resource scarcity [36]. In order to understand the magnitude of the resource requirements, resource prediction models were developed [2]. These data-driven approaches are increasingly more popular for decision-makers to make informed decisions on resource allocations affecting millions of people.

Predominantly, epidemiological models were used to predict the spread of the virus on a national and regional level. The models predicted the number of COVID-19 infections and hospital admissions, subsequently translated into bed requirements and deaths [10, 22, 24, 28, 31]. Additionally, epidemiological models were extended by incorporating lock-down measures and other non-pharmaceutical interventions to inform public policy [19]. Despite the benefits of the vast number of pandemic resource prediction models, three drawbacks limit the usefulness of these models for hospitals.

Firstly, prediction models lack the ability to integrate multiple patient flows and stochastic parameters (e.g. assumes fixed Length-of-Stay (LoS) for every patient). This reduces the accuracy and validity of the model in representing reality. However, Costa et al. [12] stated that “Using [...], average length of stay, [...] to calculate the number of critical care beds needed is mathematically incorrect because of nonlinearity and variability in the factors that control length of stay” (p.320). Additionally, Weissman et al. [45] stated that there is a need to inform the model with local data and local parameters, and to include multiple patient flows to predict resource requirements more accurately. In response, Zhang et al. [50] did model different patient flows, but failed to incorporate stochastic parameters. A systematic review of LoS parameters concluded that there is a need for stochastic LoS distributions, fitted to local and patient flow-dependent data [32].

Secondly, the prediction models concentrate on modelling a limited set of resources: (ICU) beds, ventilators and deaths. Woodul et al. [49] summarised the common strategy adopted in literature to decrease modelling complexity: “Hospital beds, [...] is used as a proxy for space, resources and providers” (p.4). Nevertheless, this approach is too simplistic and does not accurately reflect the resource scarcity in hospitals during the first wave: there were hundreds of empty beds, but there is a lack of PPE, staff, oxygen and mortuary capacity [6, 46]. Some models [37, 38] included more resources, such as PPE and staff. However, these models did not account for different patient flows (e.g. a complicated patient flow: general ward → ICU → general ward).

Finally, the overwhelming majority of the resource prediction models discussed do not predict non-COVID-19 care, whilst these activities were significantly cut down during the pandemic. This was recognised as a significant shortcoming, requiring further research [27, 30].

### Resource allocation modelling

Scarcity of hospital resources calls for effective resource allocation strategies [44], regardless of COVID-19: “Efficient functioning of a hospital depends on how it allocates its resources, particularly allocating beds to patients, a problem fraught with complexities and uncertainties” (p.298) [5]. Models were developed to evaluate resource allocation decisions, such as bed-plan expansions [11], and specific events, such as the annual winter bed crisis [43].

Literature proposed several methods to allocate scarce resources. Hospital resources can be allocated based on i) patient flows or demand intensity [8, 44], ii) priority of patient groups (e.g. by ‘ring-fencing’ capacity [1, 14]), and iii) the likelihood of favourable outcome (i.e. triage of patients [39]). In this section, we describe the limitations of models evaluating resource allocation decisions.

Firstly, disaster resource allocation models fail to provide a holistic view on health care during a disaster. Apart from resource scarcity in regular times, there is an increased scarcity of healthcare resources during disasters (incl. pandemics). However, the literature has only analysed resource allocation *within* the disaster [4, 42]. This implies that a fixed set of resources is shared between the patients affected by the disaster, rather than balanced between all patients requiring care.

Secondly, allocation models fail to acknowledge that non-pandemic care cannot be modelled as a constant baseload during a pandemic. The impact of surging pandemic care on non-pandemic care is often not evaluated in prediction or allocation models. For example, Wood et al. [48] stated that the act of balancing resources and evaluating the opportunity cost of surging capacity is left as an exercise for decision-makers. Zhang et al. [50] included regular care as a baseload factor to the model. Nevertheless, this baseload is independent of lock-down policies, the availability of resources, or the intensity of the virus-spread. The application of such a baseload is found both in models studying pandemics in general [29, 49] and COVID-19 specifically [26]. This method fails to account for essential characteristics of hospital operations during a pandemic.

### Literature gap and relevance

Based on the limitations outlined, there is a need for resource allocation models to include and balance both pandemic care and regular care [17, 27, 30]. Balancing the two types of care requires an integrated prediction of both types of care, to subsequently determine the overall impact of different allocation strategies. Secondly, there is a need to model a more comprehensive set of resources, such as staff. Thirdly, there is a need for stochastic and locally-informed parameters and processes to resemble local healthcare practises and clinical variability more closely. Subsequently, this paper aims to answer the question: What is the impact of scarce resource allocation strategies on the ability to treat patients during a pandemic?

### Structure of the paper

This paper is structured as follows. First, the modelling of patient flows and scarce resources is presented in Section 2, alongside an identification and analysis of the required input data. Following the methodology, Section 3 presents the main results of the model, tailored to a large regional hospital in England. Finally, Section 4 discusses the key findings, its implications, and limitations. Directions for further research on scarce resource allocation modelling were provided.

## Materials and methods

This study aims to quantitatively measure the impact of resource allocation strategies on the ability to treat patients. This study evaluates the specific setting of Addenbrooke’s Hospital, a major regional hospital in Cambridge (United Kingdom). However, the study is set-up to be generalisable for hospitals worldwide.

### Identification of scarce resources

The resource scarcity that hospitals face is time and context-dependent. Scarce resources were defined as: resources which i) are in greater demand than supply now or in the future, ii) are shared between COVID-19 and non-COVID-19 patients, and iii) are predictable in both supply and demand. From a long-list of eight hospital resources (i.e., beds, equipment, staff, PPE, consumables, oxygen, medication, mortuary), two sets of scarce resources were identified based on the definition provided. The definition and choice of scarce resources were co-constructed and validated by managers, planners and clinician leads in Addenbrooke’s hospital. Resources which were scarce but not shared between COVID-19 and non-COVID-19 patients were excluded, as this study aimed to explore the inter-group balancing effects.

First, critical care (CC) beds are used by both COVID-19 and non-COVID-19 patients and are extremely scarce and resource-intensive. CC is defined as the combination of the intensive care unit (ICU) and the high-dependency unit (HDU).

**Fig. 1:**
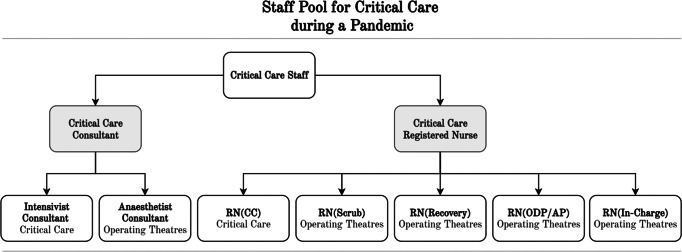
Staff pool for critical care

Secondly, the pandemic places extraordinary pressure on CC staff, requiring other staff groups to fulfill the role of CC staff, especially operating theatre staff. Hence, CC and operating theatre staff collectively form a pool of shared and scarce resources, illustrated in Fig. 1,^1^. This study focused on operating theatre staff in contrast to nursing staff from other specialties, as operating theatre staff proved to form the outer flexible deployment layer for CC at Addenbrooke’s hospital. It was assumed that all available nursing staff from other wards was already transferred to CC. Although essential to the care of patients, the other resources were assumed to have sufficient capacity.

### Mapping patient flows

This study aimed to integrate COVID-19 with forms of non-COVID-19 care. Whereas emergency department (ED) services continued throughout the pandemic, surgical care saw an inverse relationship with the COVID-19 wave: many surgeries were cancelled to enable increased capacity on CC. Hence, this study focuses on the relationship between COVID-19 and surgical patient streams.

**Fig. 2:**
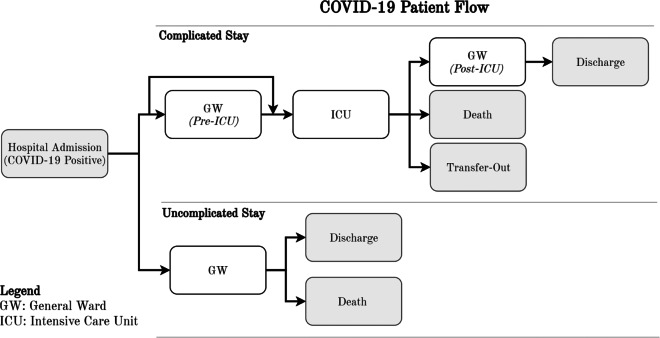
Identification of COVID-19 patient flows

Hospital patient flows are highly variable and unique. Despite the high variation, the majority of COVID-19 patients seem to follow one of two categories of patient flows (see Fig. 2): *complicated stay* (requiring GW and ICU stay) and *uncomplicated stay* (requiring GW stay only), based on experiences in Addenbrooke’s Hospital and available literature [51]. Figure 2 displays the patient flows identified by Addenbrooke’s lead infectious disease consultant, based on observed patient flows of 475 COVID-19+ patients. For simplicity, unobserved or unlikely patient flows were excluded.

**Fig. 3:**
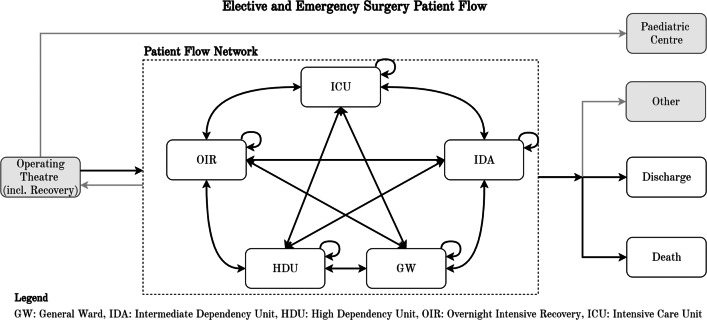
Identification of surgical patient flows

In parallel, a flow of elective (EL) and emergency (NEL) surgical patients move through the hospital after leaving the operating theatre (OR). Whereas COVID-19 patient flows were modelled using a set of *sequential-* processes, surgical patient flows were described by a process-*network* accounting for the variability in clinical needs. Figure 3 presents the network structure of levels-of-care, according to the process-network approach suggested by Devapriya et al. [16]. The network includes additional levels-of-care besides GW, HDU and ICU: the overnight-intensive recovery (OIR) and intermediate-dependency area (IDA). The modelling approach accommodates for all possible flows between all nodes, whilst allowing the transfer probability between certain nodes to be zero. To acknowledge the clinical differences between elective and emergency surgical patients, the network was modelled separately to facilitate different transfer probabilities and LoS.

### Resource allocation strategies

Three resource allocation strategies were co-constructed with directors of Addenbrooke’s Hospital: i) a proactive cancellation strategy of non-COVID-19 care, ii) a reactive cancellation strategy of non-COVID-19 care, and iii) a ring-fencing strategy of elective surgical care. The strategies reflect different ways of preparation for an uncertain wave, and vary the prioritisation of COVID-19 and elective surgical patients, which hospitals -including Addenbrooke’s Hospital- consider to deploy for a second wave (see Table 1). The first two strategies prioritise COVID-19 over elective surgery but assume different forms of preparation; the pro-active cancellation strategy enables staff training for CC whereas the reactive cancellation assumes staff training is no longer required. The ring-fencing strategy prioritises elective surgical care over COVID-19 care up to a certain threshold to enable life-saving surgery.

**Table 1:** Resource allocation strategies

	1. Pro-active	2. Reactive	3. Ring-fencing
	Cancellation	Cancellation	Theatres
Priority	1. NEL	1. NEL	1. NEL
	2. COVID-19	2. COVID-19	2. EL
	3. EL	3. EL	3. COVID-19
Close Theatres	At the start of the wave	When necessary	When necessary, up to a threshold.
Open Theatres	After peak when possible	When possible	When possible
Source	[23]	[41]	[14]

NEL: non-elective surgery, EL: elective surgery

### Key performance indicators

The resource allocation policies were analysed using a balanced set of key performance indicators (KPIs), based on hospital operations literature [16, 52]. The KPIs evaluated the core concepts: i) how many patients can be admitted to the hospital, ii) how many patients can be provided full treatment, and iii) what was the outcome of these treatments (see Table 2). ‘KPI-3 CC Rejections’ was defined as: the number of patients not being able to capture the appropriate bed or staff required for their level of care on CC [52] within a given time-frame. Rejections were documented separately from direct deaths, as rejections are the result of hospital operations rather than the patient’s clinical characteristics.

**Table 2:** Key performance indicators to evaluate resource allocation strategies

	KPI	Focus	Goal
1	Elective Surgeries	Patient arrival	Maximise
	Performed		
2	COVID-19 Admissions	Patient arrival	Maximise
3	CC Rejections	Patients deferred	Minimise
		required bed-level	
4	Deaths	Effectiveness of	Minimise
		treatment	

While acknowledging that weighting these KPIs requires ethical considerations, the *Aggregated* *Hospital* *Performance* measure (AHP) was introduced to compare the strategies using a single metric, by making the following assumptions:

- A proportion of elective surgery can be considered life-saving, avoid an -otherwise inevitable- death;
- Waiting for more than 24h for CC (i.e. CC Rejection) will result in death;
- Not admitting a COVID-19 patient will result in death.

Subsequently, the AHP is calculated using Equation 1. The ‘life-saving proportion’ was introduced based on validation of the AHP by clinicians, to account for the fact that not all surgery can be considered life-saving. A sensitivity analysis of the AHP measure is performed on the proportion of life-saving surgeries in Section 3.4. The AHP measure enables a comparison of strategies for a specific situation but should not be used as stand-alone metric to compare different hospitals or situations.

1 $$ \begin{array}{@{}rcl@{}} AHP &= & EL  Surgeries Performed  * <percent>  Life  Saving + \\ && COVID 19 Admissions  - \\ && Total Deaths - \\ && CC Rejections \end{array} $$

### Stochastic modelling

Analysing hospital patient flows and evaluating the consumption of resources requires an approach accounting for different patient flows and *variability* [12]. This section describes the modelling approach and assumptions.

#### Selection of modelling methodology

A stochastic modelling methodology is required to account for the operational and clinical variability inherent to COVID-19. The most common method to model COVID-19, *epidemiological models*, are used to predict the demand for hospital resources [10, 19, 28], but failed to model COVID-19 *and* non-COVID-19 care. On the other hand, *analytical models* are powerful to derive mathematically optimal allocation strategies for a given resource set [7]. Despite providing optimal results, fundamental assumptions limit the validity of the results in practice [7]. Finally, *simulation models* are applied for both COVID-19 and non-COVID-19 purposes [34, 35, 37, 40] by modelling patients, processes and resources [9], but have not been integrated as of yet. Seeing that COVID-19 and non-COVID-19 patients have high resource inter-dependency and diverse patient flows [3], simulation is deemed most suitable.

#### Selection of simulation paradigm

Different simulation tools have been applied to model pandemics: system dynamics (SD), agent-based modelling (ABM) and discrete event simulation (DES) [15, 18]. For this study, DES is the most suitable simulation tool. DES recognises the hospital unit-of-analysis, stochastic patient flows [21], and has the ability to analyse resource allocation policies.

#### Simulation model

The patient flows, KPIs and resource allocation strategies were implemented in Arena software, version 16 (2019, Rockwell Automation Technologies). The model is open source and modifiable but requires a commercial Arena software license. The number of replications was determined using the Monte-Carlo sampling method [47]. The model was run for 85 replications to account for a desired error margin of 1 CC bed at a 95% confidence level. The output was analysed and visualised using Python, version 3.7 (2020, Python Software Foundation). The results were supplemented with 5% and 95% percentile bands to represent the degree of uncertainty.

#### Modelling logic

**Fig. 4:**
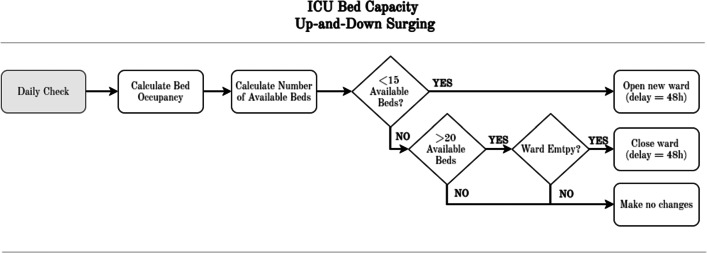
Capacity surge modelling Intensive Care Unit (ICU)

The model encompasses three main capacity decision-making heuristics derived from hospital operational processes, co-constructed and validated by hospital directors and clinicians. Firstly, **ICU capacity surging** is potentially required if too many (COVID-19) patients require an ICU bed. When and how much to expand is detailed in the ICU capacity surge logic, presented in Fig. 4.

**Fig. 5:**
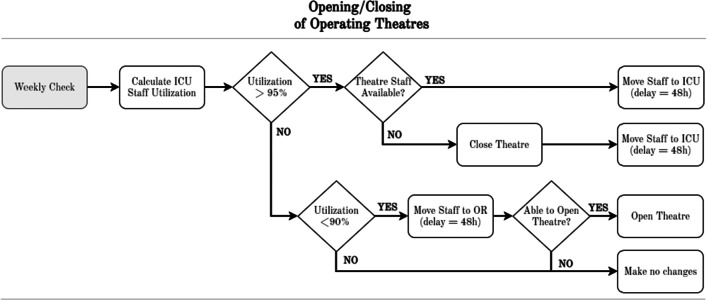
Modelling of opening and closing of operating theatres. OR: operating theatre/room, ICU: intensive care unit

Secondly, Fig. 5 illustrates the process of **opening/closing theatres** to account for additional staff needs on CC during a pandemic wave. Opening/closing theatres is evaluated weekly, to account for the preparation of theatres, equipment, staff and patients for these major operational changes.

**Fig. 6:**
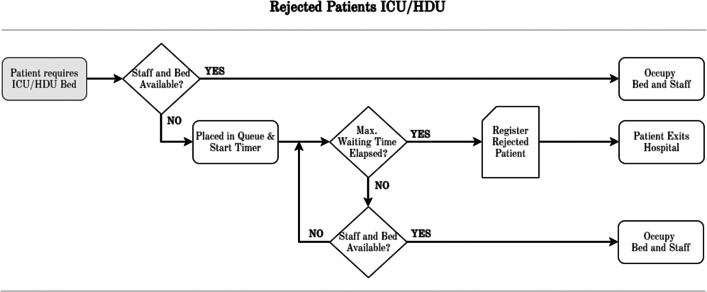
Intensive Care Unit (ICU) and High Dependency Unit (HDU) patient rejection modelling

Thirdly, there may be insufficient beds or staff available for patients requiring ICU/HDU, resulting in **CC rejections**, illustrated in Fig. 6. Following the definition of KPIs in Section 2.4, rejections and deaths were documented separately to enable in-depth analysis.

### Data collection and processing

The collection and processing of the input data is discussed in the subsequent sections, following the patient’s journey: arrival scenarios, hospital patient flows, LoS and resource consumption.

#### Patient arrivals & scenarios

**Fig. 7:**
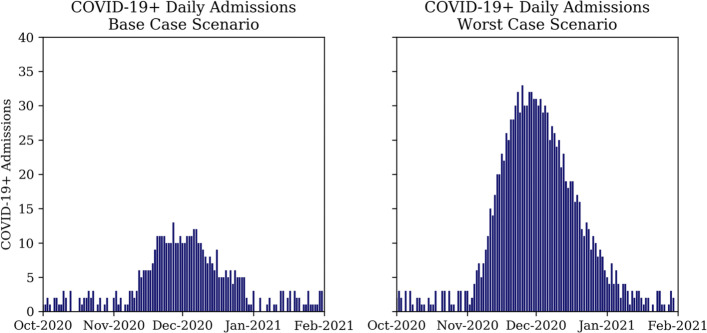
COVID-19 daily admission scenarios; base case (left) and worst case (right)

The simulation model evaluates the resource allocation strategies for two COVID-19-*positive* hospital admission scenarios, depicted in Fig. 7. First, the base case scenario describes hospital admissions to Addenbrooke’s hospital similar to what was observed during the first wave. Second, the worst case scenario describes an alternative scenario to the base case scenario, with a peak number of admissions more than twice as high.

**Fig. 8:**
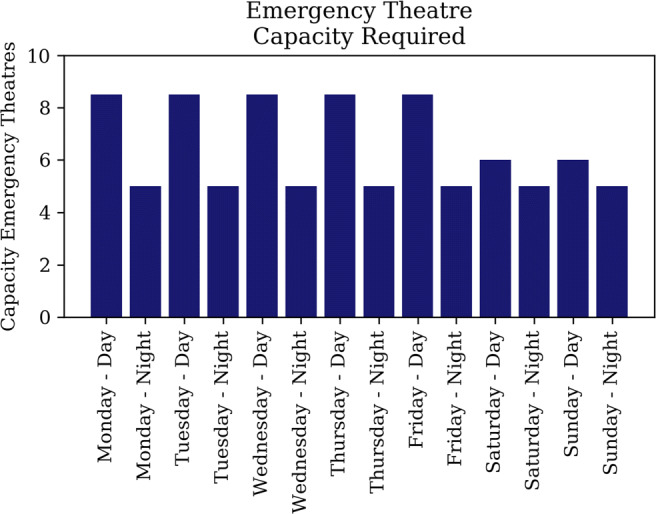
Emergency theatre capacity Addenbrooke’s

The arrival of elective and emergency surgical patients is determined separately. Figure 8 illustrates the capacity of emergency theatres required by Addenbrooke’s hospital to treat all emergency surgical patients within appropriate time-frames. The number of daily elective surgeries is based on the number of elective theatres open, and assumed to have ample demand, seeing the insurmountable backlog [13, 20].

#### Transition probabilities

After *COVID-19 patients* enter the hospital according to the arrival schedule, they follow one of the patient flows depicted in Fig. 2, with the transition probabilities presented in Table 3, based on anonymised patient flow data of 475 COVID-19 patients.

**Table 3:** COVID-19 transfer probabilities based on patient data March-June 2020, Addenbrooke’s Hospital

Transfer Probability	Value
Survival Rate ICU	61.1%
Survival Rate GW	71.8%
ICU Required	22.4%
GW (Pre-ICU) Required	28.3%
Transfer-out Probability	7.4%

GW: general ward, ICU: intensive care unit

In parallel, *surgical patients* are released from the operating theatre and enter the hospital patient-flow network (Fig. 3). The patient-flow network is accompanied by a transfer-probability matrix, which was constructed separately for elective and emergency surgical patients (Tables 4 and 5, respectively). The matrices are derived based on 28,831 patients receiving surgery in 2019, by analysing their 128,811 anonymised and unique one-step ward transitions at Addenbrooke’s hospital, using a three-step methodology:

1. Every patient’s individual sequence of ward locations is transformed into a patient flow of ‘level-of-cares’ (e.g. GW → OR → OIR → ICU → GW).
2. Each individual patient flow is split into a set of one-step transitions (e.g. GW → OR; OR → GW).
3. Based on all one-step ward transitions, the probability of transitioning to another ward is calculated, conditioned on the current location.

It is important to note that this approach does take into account a patient’s previous level-of-care when determining the probability of transferring, but is limited by not considering *all* previous levels-of-care of a patients journey. This reduces the accuracy of predicting patient flows for the surgical patients.

**Table 4:** Transition probability matrix for elective surgical patients

Elective		Destination								
Transition Prob. (in %)		OR	ICU	OIR	HDU	IDA	GW	Death	Discharge	Other
Origin	OR	0.10	0.69	3.82	0.05	0.30	91.3	0.00	0.04	3.66
	ICU	16.8	3.29	0.00	0.41	19.7	50.2	2.47	4.94	2.06
	OIR	1.84	1.84	0.00	0.25	29.2	56.9	0.00	2.82	6.99
	HDU	0.00	0.00	0.00	0.00	0.00	81.8	0.00	18.1	0.00
	IDA	4.38	2.84	0.00	0.26	0.00	88.4	0.00	1.80	2.32
	GW	1.21	0.40	0.48	0.04	0.23	4.21	0.04	92.7	0.64

**Table 5:** Transition probability matrix for emergency surgical patients

Emergency		Destination								
Transition Prob. (in %)		OR	ICU	OIR	HDU	IDA	GW	Death	Discharge	Other
Origin	OR	0.78	15.0	0.40	1.37	1.77	75.5	0.22	0.02	4.91
	ICU	23.3	2.95	0.00	10.19	9.39	42.8	6.69	3.03	1.59
	OIR	1.89	3.77	0.00	1.89	15.0	71.7	0.00	1.89	3.77
	HDU	9.88	6.48	0.00	0.00	0.00	82.4	0.62	0.62	0.00
	IDA	9.12	7.06	0.00	0.29	0.00	78.5	1.76	2.65	0.59
	GW	9.24	2.88	0.33	1.38	1.32	29.7	0.86	53.3	0.94

#### Length-of-stay

Demand for resources by *COVID-19 patients* is significantly influenced by the LoS of patients, and hence the accuracy of resource demand-prediction models are sensitive to the parameters used to model the LoS distribution [32]. LoS distributions were fitted for each level-of-care (e.g. ICU) on anonymised patient flow data. More specifically, the stage in the patient flow is incorporated. For example, the average time on a general ward before going to ICU is significantly shorter than the time spent on a general ward after being discharged from ICU [51].

The distribution fitting was performed using @RISK software, version 8 (2005, Palisade USA), using the patient data of 475 COVID-19+ adult patients admitted to Addenbrooke’s hospital between March-June, 2020. The best fit was determined based on a combination of the Chi-Squared and Kolmogorov-Smirnov (KS) test.

For *surgical patients*, the LoS was constructed based on analysing the anonymised patient flows of 28,831 patients receiving surgery in 2019 at Addenbrooke’s hospital. To account for LoS differences based on improving and deteriorating health-conditions, the LoS was based on both the level-of-care of interest and the preceding level-of-care [16], similar to the methodology described in Section 2.6.2. To enable the health condition-based LoS, the simplifying assumption was made that the LoS can be modelled in a deterministic manner for surgical patients, using the average LoS.

#### Resource consumption

**Fig. 9:**
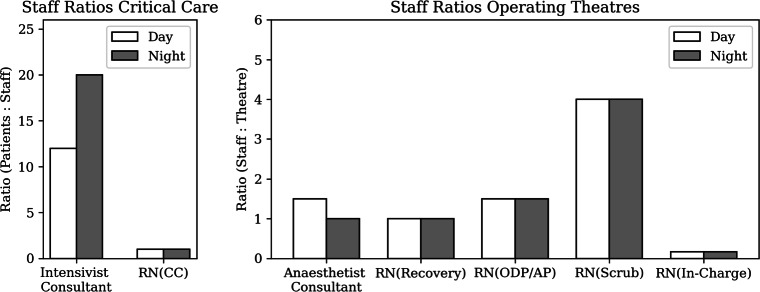
Staffing ratios for each department in Addenbrooke’s

A patient requires both a bed and staff. Staff as a resource is expressed in whole time equivalent (WTE), which enables a capacity management on a weekly level. Figure 9 presents the estimated staffing ratios in Addenbrooke’s hospital during a pandemic. Additionally, the model captures the ability to work together with the Independent Sector (IS). IS theatres only require a hospital’s anaesthetist consultant, but no theatre nurses. Finally, 25% of staff capacity was reserved for annual leave, sickness, shielding and training.

Also *non-surgical patients* occupy CC resources, usually through direct referral from the ED. This additional patient flow to CC is accounted for by including a baseload-factor, derived from historical CC occupancy. Data analysis suggests on average 15 patients in ICU and 10 in HDU, are non-surgical patients.

### Model validation

Model validation is performed to evaluate the degree to which the model accurately represents reality [25].

Firstly, **black-box testing** is the validation of the model output with the actual numbers observed in reality. Black-box testing was performed by comparing the predicted and actual bed occupancy for COVID-19 during the first wave, and by comparing the predicted number of theatres open and the actual number of theatres open.

Secondly, **structure-verification testing** was applied [25] to validate the structure and processes in the model [52]. More specifically, patient flows, decision nodes and main decision-making heuristics were validated by operational managers and clinicians.

## Results

The impact of resource allocation strategies on hospital performance was evaluated using the proposed simulation model. This chapter highlights the results from data-gathering and processing, and the results from the simulation model.

### Data analysis results

#### Patient flow transition probabilities

A COVID-19 patient follows one of a set of structured flows (see Fig. 2), for which the probability of following an arbitrary route was presented in Table 3, based on anonymised patient flow data. On the other hand, a surgical patient’s journey is driven by their health condition. The transition matrices for elective and emergency surgery (Tables 4 and 5, respectively) present the chance of transferring to a specific location (i.e. destination), given the previous location (i.e. origin), after completing the LoS of the previous location. It is also possible for a patient to transition to a destination with the same level-of-care as the origin, e.g. a patient moving from a specialised colorectal post-surgical recovery ward (GW) to a general oncology ward (GW). Hence, *P*(*Destination* = *Y* |*Origin* = *Y* ) ≥ 0, where *Y* represents any level-of-care.

The results show that a patient will always have the highest chance to move to a general ward or to get discharged from a general ward. Also, the chance of transitioning from operating theatres to CC is significantly higher for emergency (16.4%) than for elective (0.74%) surgical patients.

#### Length-of-stay

##### COVID-19 length-of-stay

**Fig. 10:**
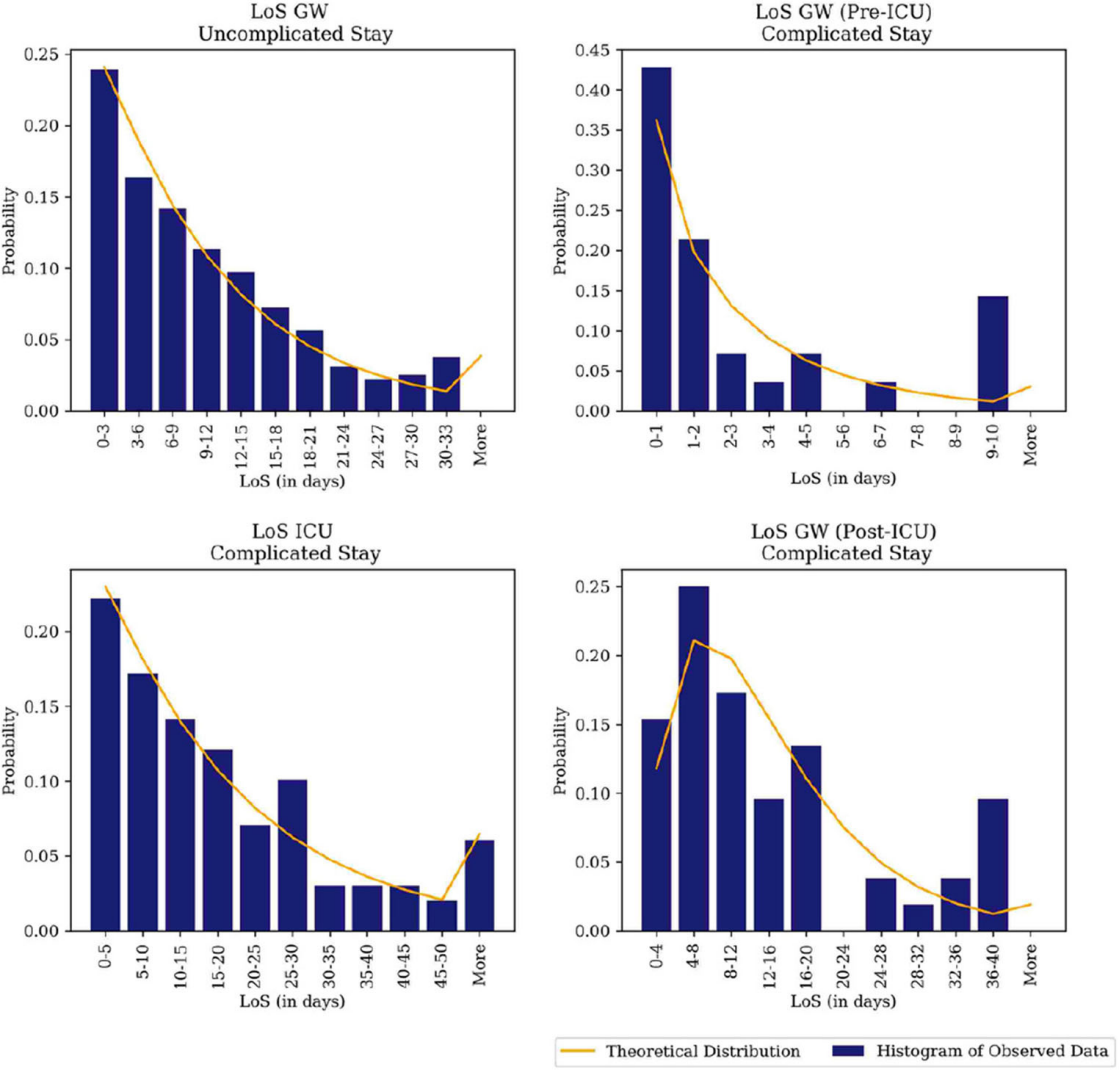
Length of Stay (LoS) distribution fitting COVID-19 Patient Flows. GW: General Ward, ICU: Intensive Care Unit

Table 6 presents the best-fitted distribution to capture the variability in LoS of COVID-19 patients. The null-hypothesis (i.e. the theoretical distribution accurately reflects the empirical distribution) was accepted at 5% significance-levels (i.e. *p*-value > 0.05) for each of the fitted distributions. Figure 10 illustrates the high variability in LoS and the visual fit of the fitted theoretical distributions.

**Table 6:** Length of Stay (LoS) distribution fitting COVID-19 Patients. GW: General Ward, ICU: Intensive Care Unit

Parameter	n	Mean (days)	SD	Distribution	Chi-Sq.	KS	p-value
GW (Pre-ICU)	28	2.65	3.13	Gamma(*α*= 0.81, *β*= 3.23)	*χ*^2^(27)= 4.14	0.19	0.313
ICU	99	18.4	17.9	Weibull(*α*= 1.02, *β*= 18.6)	*χ*^2^(98)= 12.8	10.0	0.131
GW (Post-ICU)	52	13.6	11.2	Erlang(*m*= 2, *β*= 6.80)	*χ*^2^(51)= 13.0	3.00	0.054
GW (Uncompl.)	346	10.3	9.61	Weibull(*α*= 1.03, *β*= 5.41)	*χ*^2^(345)= 12.2	18.0	0.662

##### Post-surgical length-of-stay

Tables 7 and 8 present the average LoS for each origin-destination combination for elective and emergency patients, respectively. The results reiterated the need for origin-dependent LoS parameters: e.g. the mean LoS for elective patients on GW and ICU ranged between 0.9-8.1 and 3.3-5.4 days, respectively.

**Table 7:** Average LoS for elective surgical patients

Elective		Destination				
LoS (in days)		ICU	OIR	HDU	IDA	GW
Origin	OR	4.46	1.13	1.54	2.39	0.85
	ICU	3.30	0.00	0.94	3.34	7.55
	OIR	4.05	0.00	3.43	3.15	5.46
	HDU	0.00	0.00	0.00	0.00	1.95
	IDA	5.43	0.00	1.05	0.00	8.10
	GW	3.29	1.08	4.22	3.16	2.68

**Table 8:** Average LoS for emergency surgical patients

Emergency		Destination				
LoS (in days)		ICU	OIR	HDU	IDA	GW
Origin	OR	5.81	1.16	3.46	2.19	4.78
	ICU	3.28	0.00	6.07	2.99	9.32
	OIR	2.67	0.00	2.68	4.00	4.01
	HDU	4.37	0.00	0.00	0.00	6.93
	IDA	4.66	0.00	0.47	0.00	8.75
	GW	3.54	0.91	2.74	2.57	5.55

### Model validation

**Fig. 11:**
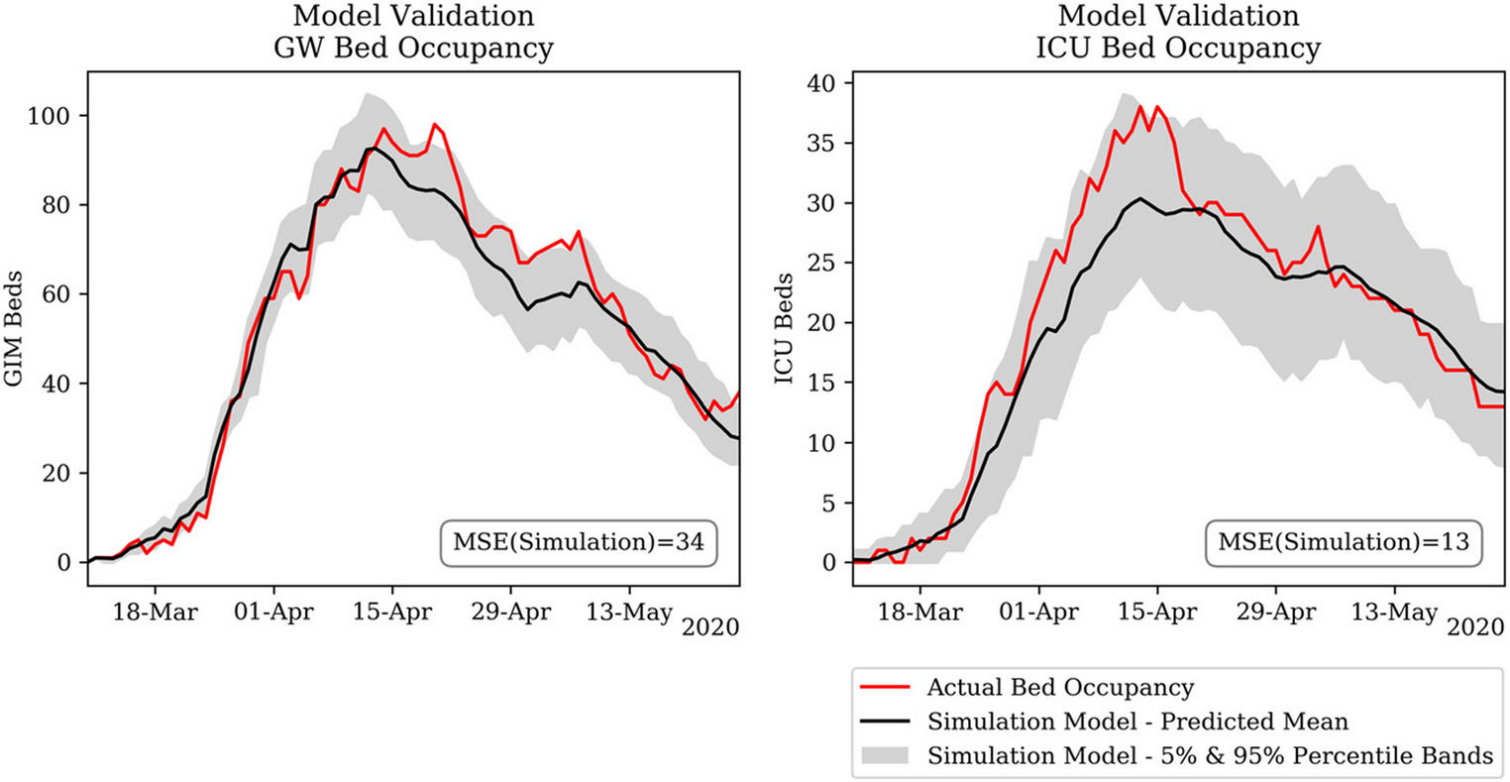
Black-box testing of COVID-19 General Ward (GW) (left) and Intensive Care Unit (ICU) (right) Bed Occupancy

Black-box testing was performed to evaluate the accuracy of model predictions with the actual observations in the hospital. First, black-box testing for COVID-19 bed occupancy was performed over the period March - June 2020, based on actual admissions. Figure 11 illustrates the high accuracy of the model in terms of GW bed occupancy and ICU bed occupancy.

**Fig. 12:**
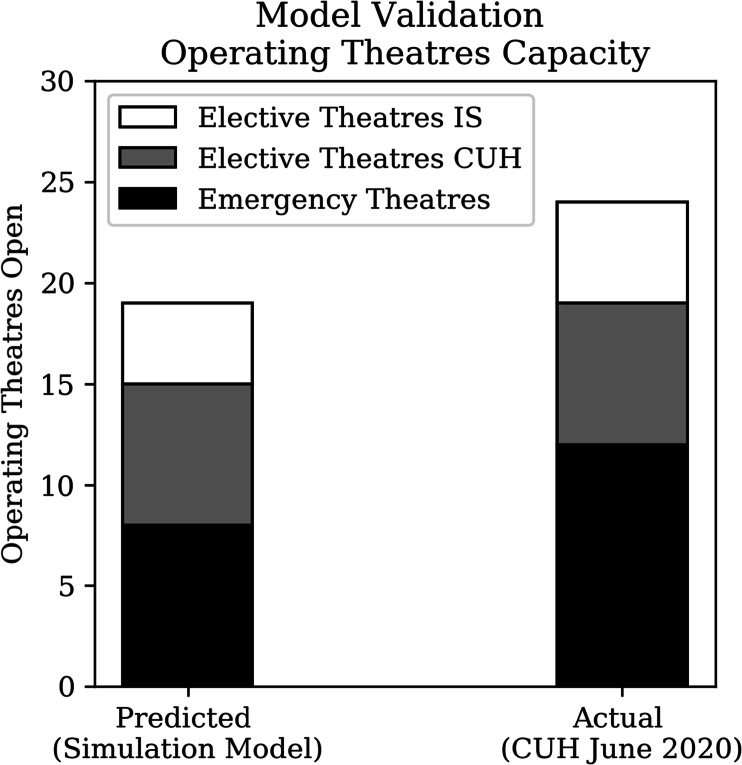
Black-box testing of Operating Theatre Capacity. CUH: Cambridge University Hospitals, IS: Independent Sector

On the other hand, Fig. 12 shows that the model underestimates the total number of operating theatres which can be opened during an *in-between-waves* context (i.e. June 2020) by 20%. Two assumptions concerning staff requirements explain the discrepancy. Firstly, ‘COVID-19 staffing ratios’ were assumed for all operating theatres (i.e. increased staffing ratios) to account for infection-control regulation. However, in June 2020 several ‘normally-staffed’ theatres were in operation, due to the low prevalence of COVID-19 in the hospital and community. Secondly, the model assumed a COVID-19 consultants rota, requiring more consultants to be present in the hospital during the night, reducing the available capacity for elective theatres.

### Resource allocation strategy results

The simulation model enabled an analysis of the resource allocations strategies. This section presents the in-depth results for the individual strategies, followed by an overall comparison.

#### Resource allocation strategies

##### Strategy 1 - proactive cancellation of elective surgery

**Fig. 13:**
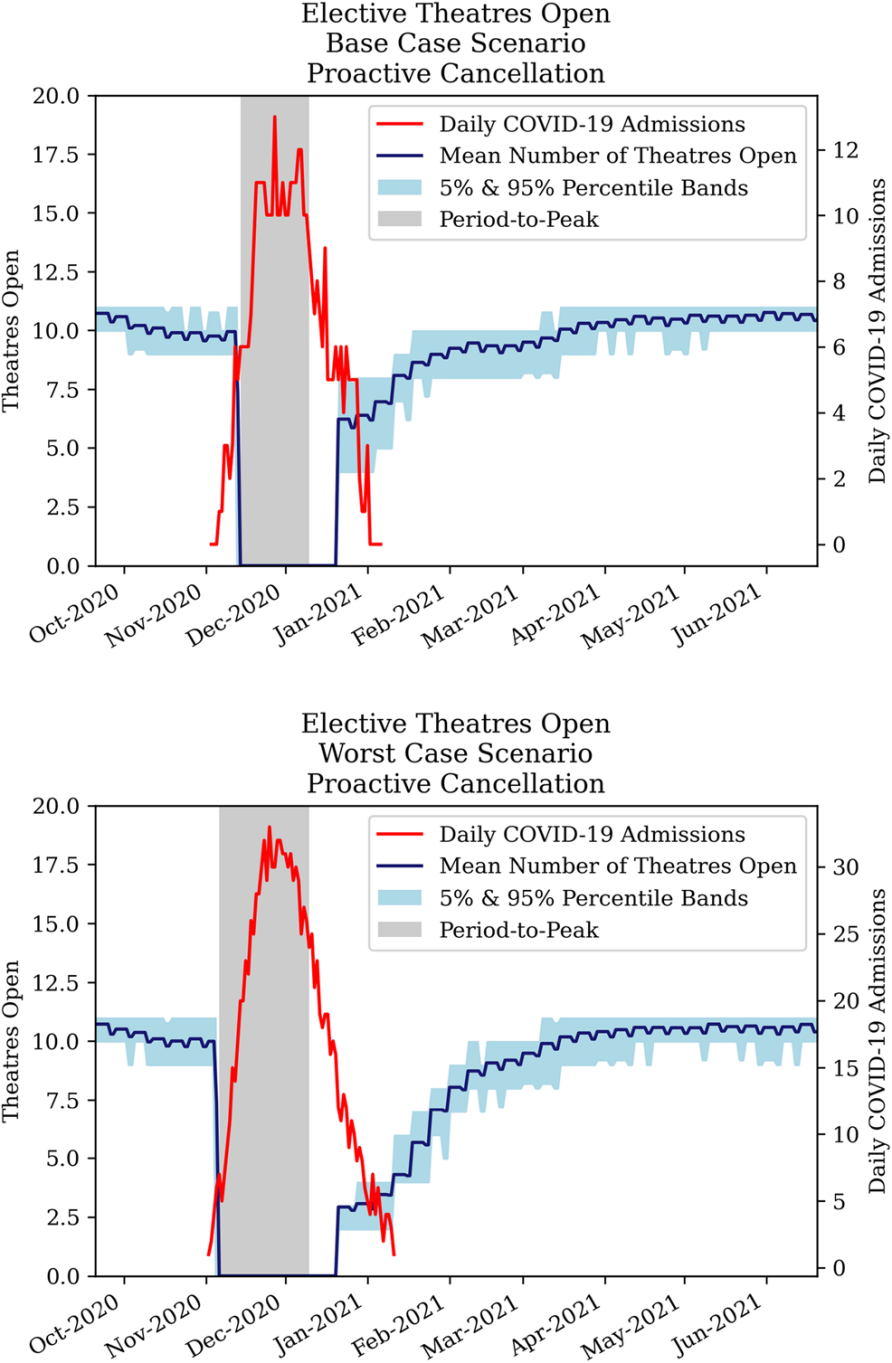
Development of elective theatres open under proactive cancellation policy for COVID-19 base case (top) and worst case (bottom)

The first strategy dictates that from the onset of a COVID-19 wave, all elective surgery is cancelled, such to allow for CC training for theatre staff. Figure 13 illustrates the evolvement of open elective theatres over time, showing a significant drop in capacity under both the base case and worst case COVID-19 scenarios. In a worst case scenario, the process of opening theatres is more gradual and takes an additional month to achieve the same level, compared to a base case scenario. Notably, Fig. 13 refutes the idea that the end of the admissions peak inherently marks the start of opening theatre capacity. Firstly, the evaluation of opening capacity occurs weekly and takes a subsequent period to transition. Secondly, the CC occupancy graph does not necessarily match the COVID-19 admissions graph.

##### Strategy 2 - reactive cancellation of elective surgery

**Fig. 14:**
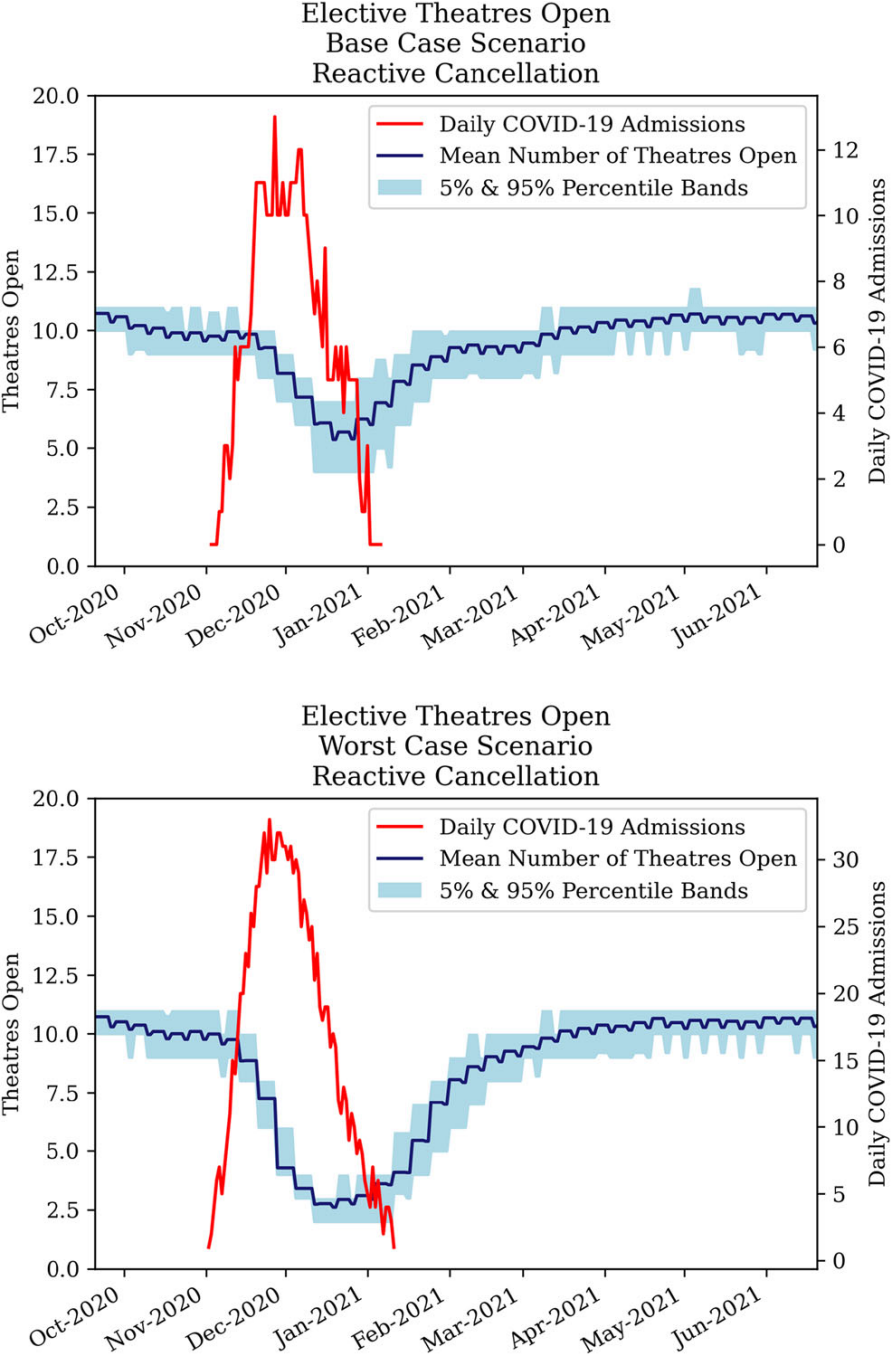
Development of elective theatres open under reactive cancellation policy for COVID-19 base case (top) and worst case (bottom)

The second strategy is similar to the proactive cancellation strategy, but elective theatres are opened or closed in a more agile way. Figure 14 illustrates that 60% more theatres need to be closed during a worst case scenario than in a base case scenario. The results suggest that Addenbrooke’s hospital can always maintain some level of elective surgery throughout the pandemic. Finally, the recovery period for both scenarios takes approximately 4-5 months, affecting elective capacity until April 2021.

##### Strategy 3 - ring-fencing elective surgery

**Fig. 15:**
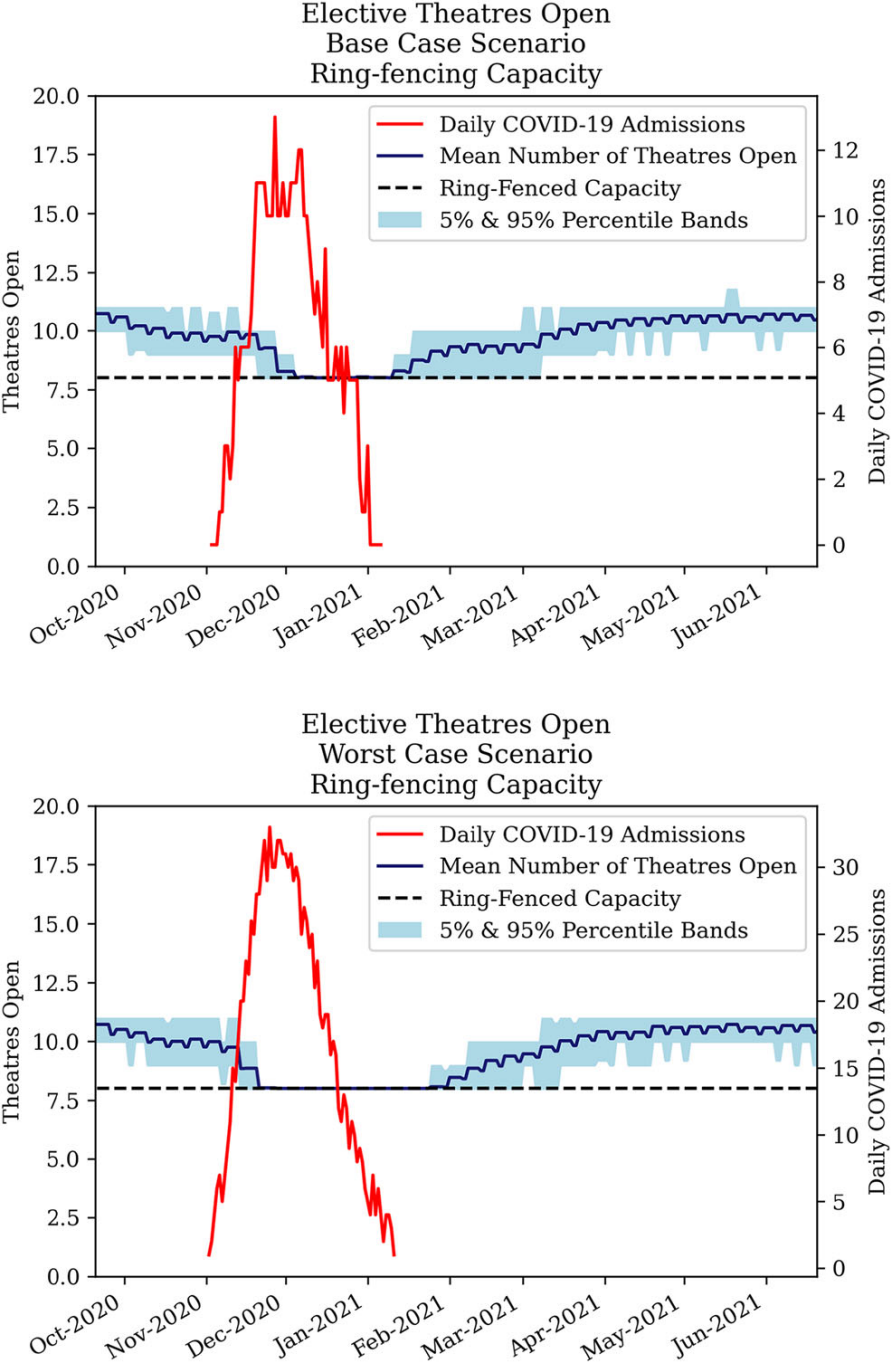
Development of elective theatres open under ring-fencing policy for COVID-19 base case (top) and worst case (bottom).

The ring-fencing strategy limits the process of closing theatres to a certain level to facilitate elective surgery. Figure 15 shows that for a base case scenario, ring-fencing resulted in relatively minor differences compared to a reactive cancellation strategy. However, in a worst case scenario, significantly more theatres remain open. The secondary effects were analysed in subsequent sections.

#### Comparative analysis

The previous section presented the impact of resource allocation policies on the ability to open elective theatres. A comparative analysis of strategies aids the discussion to determine the ‘optimal’ resource allocation strategy.

##### Hospital front-end

**Fig. 16:**
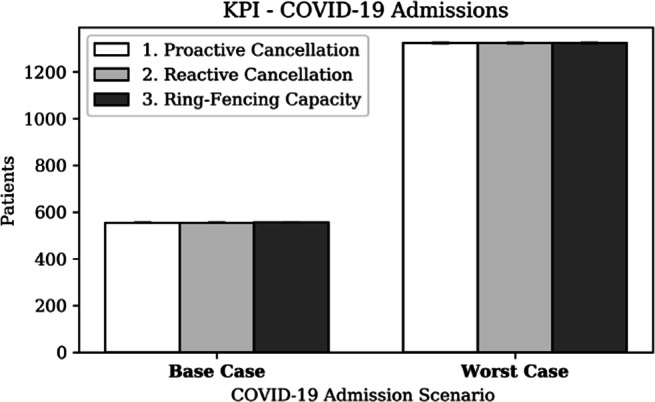
KPI - COVID-19 admissions

**Fig. 17:**
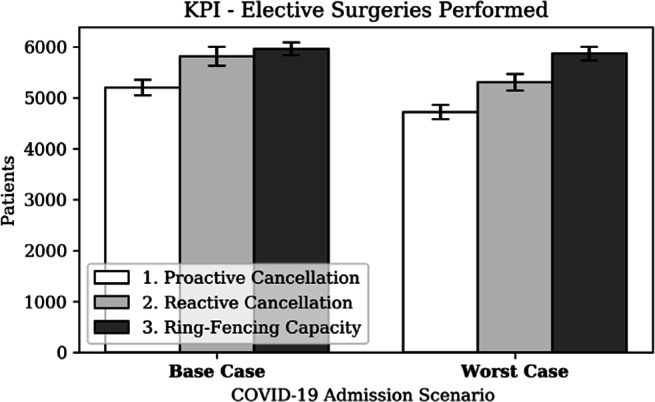
KPI - elective surgeries performed

Hospitals aim to admit any patient requiring care without delay. Figure 16 presents the number of admitted COVID-19 patients and Fig. 17 presents the number of elective surgeries performed, for each strategy, grouped by COVID-19 scenario. The negligible effect of the strategies on COVID-19 admissions is explained by the assumption that every patient requires a GW stay before potentially requiring CC. It was further assumed that GW beds are not scarce in a hospital during a pandemic, in line with Addenbrooke’s hospital’s experience during the first wave.

The ability to perform surgery is significantly dependent on the strategy. Firstly, proactive cancellation results in 11% fewer surgeries compared to reactive cancellation, as an effect of proactively closing theatres. Secondly, a ring-fencing strategy translated in significantly (i.e. 10%) more surgeries compared to a reactive strategy in a worst case scenario, but only performs marginally better (i.e. 2.5%) in a base case scenario.

##### Patient outcomes

**Fig. 18:**
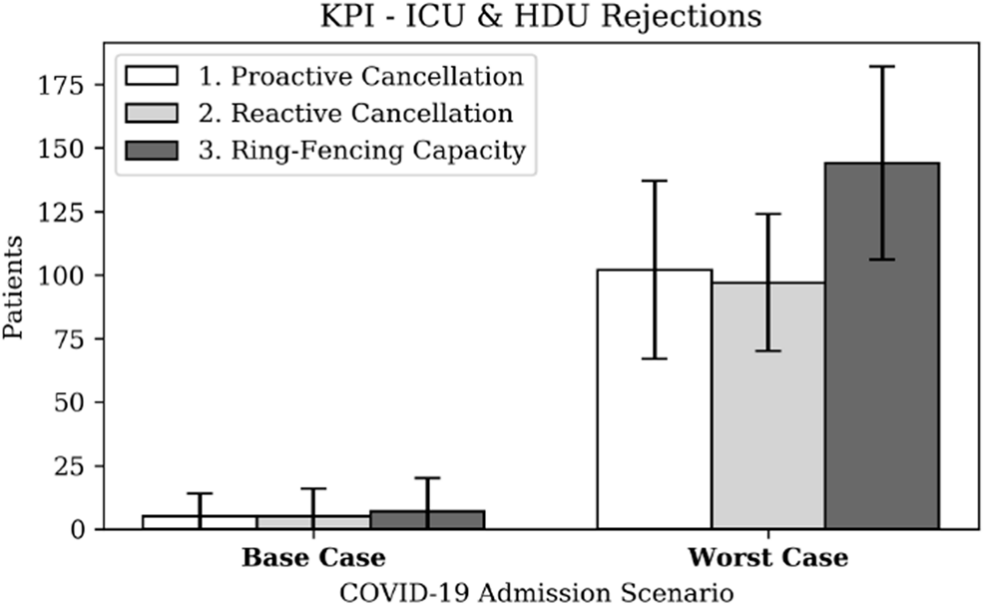
KPI - ICU & HDU rejections. ICU: intensive care unit, HDU: high dependency unit

Even though hospitals aim to maximise the number of people it can admit, it simultaneously aims to maximise patient treatment and outcomes. Figure 18 presents the number of rejections for each strategy and scenario. The most important conclusion is that the ability to treat patients is greater under a base case scenario; a worst-case scenario results in at least 20 times more CC rejections. Moreover, the ring-fencing strategy results in 10% more surgeries but 50% more rejections. These results expose the multi-objective optimisation context: maximising the number of surgeries performed whilst minimising the number of rejections.

**Fig. 19:**
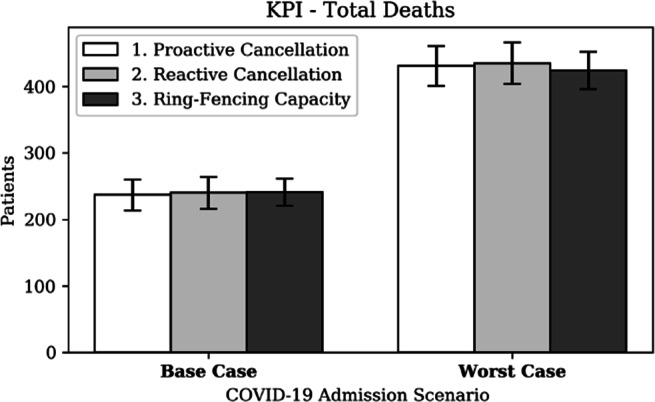
KPI - Total direct deaths (excl. CC rejections and surgeries performed)

Minimising the total number of deaths is often a core objective during pandemics. This study defined deaths more holistically by including non-COVID-19 deaths; specifically surgical patients not surviving their hospital stay. Figure 19 shows that the total direct deaths are twice as high in the worst case scenario. While there is no significant difference in a base case scenario, a ring-fencing strategy results in slightly fewer direct deaths in a worst case scenario. This effect is predominantly explained by the increased rejection of COVID-19 patients under a ring-fencing strategy, which results in fewer *direct* deaths in the hospital.

##### Aggregated hospital performance

**Fig. 20:**
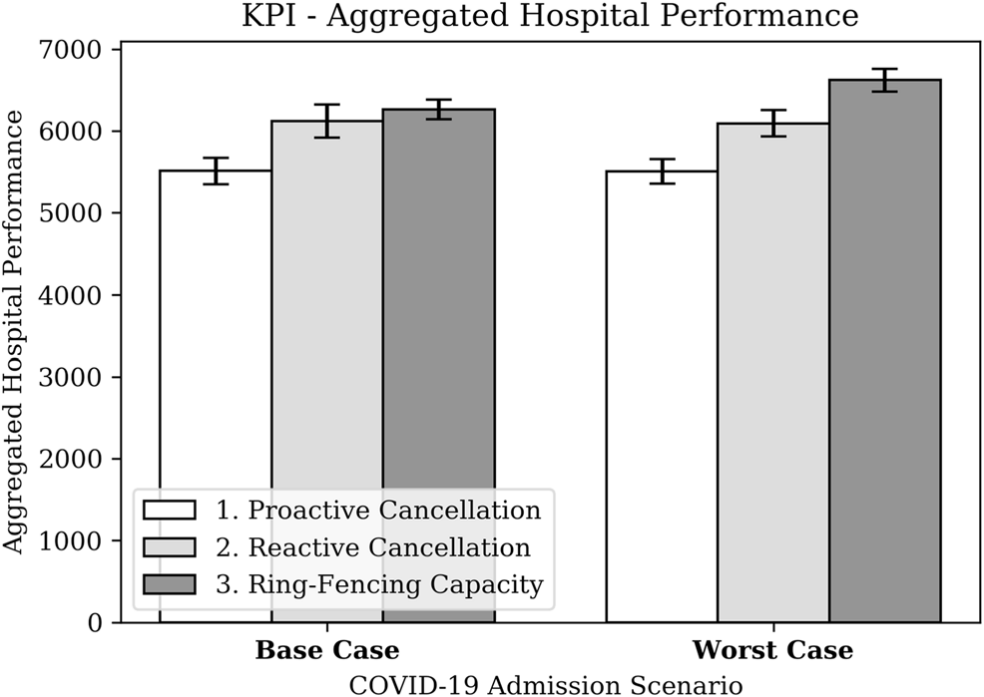
KPI - Aggregated Hospital Performance (AHP)

The four KPIs were aggregated by the AHP-measure into a single-currency metric by using the assumptions stated in Section 2.4. In addition, for this preliminary analysis, we assume that 100% of the elective surgeries are life-saving, e.g., the unavailability of surgery will result in the death of the patient. Figure 20 presents the AHP results, suggesting that a ring-fencing strategy is superior over both the proactive and the reactive cancellation strategy, resulting in a 2-20% performance improvement. A proactive cancellation strategy -potentially unnecessarily- reduces the number of surgeries performed, where the reactive cancellation policy prioritises resources to a group of resource-intensive patients with a relatively low likelihood of a favourable outcome. Both the order of magnitude of average AHP and the ranking of strategies are consistent and independent of the COVID-19 scenarios. Hence, the AHP-measure is robust in different contexts.

### Sensitivity analysis

**Fig. 21:**
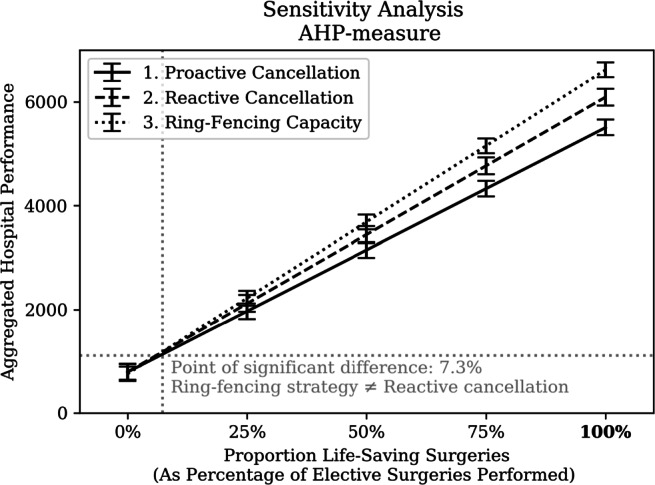
Sensitivity analysis of Aggregate Hospital Performance (AHP)-measure on proportion of surgeries classified as life-saving

A sensitivity analysis was performed to evaluate the robustness of the model and results. More specifically, the underlying assumptions of the proposed AHP-measure were evaluated. The proportion of elective surgeries which can be considered ‘life-saving’ was stretched between 0-100%. Figure 21 shows the sensitivity analysis of performance of the resource allocation strategies in terms of AHP. The null-hypothesis that a ring-fencing strategy outperforms a reactive cancellation strategy was rejected if only less than 7.3% of the elective surgeries can be considered ‘life-saving’ (*α* = 5*%*). Otherwise, the ring-fencing strategy prioritising elective surgical care seems superior.

## Discussion

### Key findings

This study evaluated the impact of resource allocation strategies on the ability to treat patients. First of all, it can be concluded that a single COVID-19 wave significantly impairs the hospital’s ability to treat patients for 4-5 months. COVID-19 patients have a higher probability of requiring CC (20% vs < 1%), a significantly longer LoS (19 vs 4 days), and a significantly higher probability of dying on CC (36% vs 2.75%) compared to elective surgical patients.

Secondly, a *proactive cancellation strategy* enables staff training for CC and allows hospitals to prepare for the ‘unknown’ influx of COVID-19 patients. However, this does come at the cost of 11% fewer surgeries compared to a reactive cancellation strategy, while not resulting in fewer death or CC rejections.

Under a *reactive cancellation strategy* where a staff training period is not required, significantly more surgeries can be performed. As a result, this strategy enables more surgery without seeing increased CC rejections or deaths.

Thirdly, a *ring-fencing strategy* for operating theatres enables surgical capacity regardless of COVID-19, translating in 2.5-10% more surgeries compared to a reactive strategy for a base case and worst case scenario, respectively. However, CC will be unable to cope with the influx of COVID-19 patients, especially under a worst case scenario; resulting in 50% more CC rejections.

Overall, no strategy outperforms on all aspects. To consolidate the different KPIs, the AHP-measure was introduced. The measure is dependent on the assumptions that all surgery is life-saving surgery during a pandemic, and that not admitting a COVID-19 patient results in death. According to the AHP-measure, a ring-fencing strategy achieves an average AHP improvement of 12% over the other strategies, potentially saving more lives. The increased number of CC rejections under a ring-fencing strategy is outweighed by the vast amount of additional surgeries performed. Such finding importantly goes against the strategy adapted by hospitals worldwide: prioritising COVID-19 patients. The dominance of the ring-fencing strategy in terms of AHP is explained by the fact that COVID-19 is a resource-intense disease; occupying resources for a significant amount of time with a relatively low likelihood of favourable patient outcomes.

Finally, the sensitivity analyses on the AHP-measure showed that only a small proportion of surgeries (> 7.3%) have to be considered ‘life-saving’ to achieve a significant difference between reactive cancellation and ring-fencing strategies in favour of ring-fencing non-COVID-19 surgical care over COVID-19. Further work is required on the life-saving nature of elective surgeries before any conclusive remarks can be made. Furthermore it could be argued that a truly life-saving situation would be treated under emergency surgery, which was outside the remit of this study.

### Implications

The key findings lead to several implications for hospitals on the modelling of resource utilisation, the preparation for a second wave, and on the allocation of resources during a pandemic. Firstly, the results confirm the need for *stochastic* and *integrated modelling* of COVID-19 and non-COVID-19 care. Secondly, resource prediction models should provide predictions on a wider range of resources, including CC nurses and consultants, to enable capacity management on the *actual bottlenecks*.

Besides the implications for modelling, this study also has implications on the *prioritisation of COVID-19 patients*. COVID-19 is a resource-intense disease with a relatively low likelihood of a favourable outcome. A significant trade-off exists between COVID-19 and surgical patients. A ring fencing strategy seems to outperform the other strategies as long as more than 7.3% of surgeries are life-saving. In conclusion, this evaluation aids an ethical discussion on the prioritisation of patients and its effects.

Finally, this evaluation demonstrated the need for hospitals to engage in *preparation* by training staff for CC and improving organisational flexibility. Also, hospitals can engage in resolving bottlenecks, e.g. by recruitment efforts. This will enable hospitals to maximise the number of patients, both COVID-19 and non-COVID-19, they can treat during the pandemic.

### Limitations

This study made several assumptions limiting the accuracy of the model. Firstly, the LoS for surgical patients was not stochastically modelled using probability distributions. Additionally, a patient’s journey was not modelled on an individual’s clinical data and surgical procedure (i.e. patient waiting list database) but based on historical data for every origin-destination combination.

Secondly, even though this evaluation made a first attempt in modelling COVID-19 *and* non-COVID-19 patients, several patient streams were still approximated using a baseload. Moreover, this study evaluated resources beyond beds, but assumed sufficient capacity for other essential resources, such as ventilators and PPE. Resources such as PPE were extremely scarce during the first wave, and it should be evaluated if these resources will again form a bottleneck.

Finally, the *AHP-measure* enables a single-currency comparison of strategies but makes fundamental assumptions limiting the validity of the aggregate measure. First of all, even though all surgeries classified as ‘P2’ according to the Royal College of Surgeons of England clinical prioritisation rubric should be performed within 30 days to prevent life altering/threatening consequences [33], those patients do not necessarily die if surgery is postponed. Therefore, this medical classification does not necessarily accurately reflect the ‘proportion of life-saving surgery’. Secondly, not admitting a COVID-19 patient does not necessarily result in death. Hence, it is important that the AHP-measure is considered with some degree of reservations, and that the in-depth KPIs are consulted to evaluate the performance of a strategy comprehensively.

### Future research

This study is the first in modelling the effect of allocating resources between pandemic and non-pandemic patients. In order to overcome the limitations and continue to explore how patient outcomes can be maximised, several recommendations for future research are proposed.

First of all, new *treatments for COVID-19*, like remdevesir, have an impact on the patient flow, LoS and patient outcomes. Therefore, research is recommended to evaluate how these treatments will result in reduced resource requirements and increased capacity to treat non-COVID-19 patients. It is expected that new treatments for COVID-19 will reduce the strain on resources and increase the favourability of prioritising COVID-19 care.

Secondly, this study recognised the *interdependency of hospital services*, such as CC and operating theatres. However, CC and operating theatres are only two areas affected by COVID-19. General wards are similarly impacted and potentially require additional staff during a second wave. Hence, further research is suggested i) to evaluate which areas are most significantly affected and ii) to include these areas in the model, to more comprehensively analyse the impact of resource allocation strategies.

Thirdly, the results of the AHP-measure suggest that COVID-19 is a resource-intensive disease with a relatively low likelihood of a positive outcome, advocating for more life-saving surgery. The AHP-measure requires an *ethical evaluation* to determine how deaths, rejections, admissions and surgeries can be combined while accounting for factors, such as i) likelihood of positive treatment outcome, ii) resource intensity of treatment, iii) potential harm of postponing treatment.

## Conclusion

As part of Addenbrooke’s Hospital operational response to the pandemic, this evaluation aimed to answer the question: What is the impact of scarce resource allocation strategies on the ability to treat patients during a pandemic? The main findings show that a proactive cancellation strategy enables staff training, but reduces a hospital’s ability to perform surgery by 11% while not significantly reducing deaths or rejections compared to a reactive cancellation strategy. Moreover, a ring-fencing strategy outperforms all other strategies in terms of surgeries performed and total deaths, but at the cost of 50% more CC rejections.

When evaluating the performance of the strategies using the AHP-measure, this study suggests that prioritising elective surgery over COVID-19 if a hospital sees a high proportion of life-saving surgeries could lead to better outcomes overall even though it might lead to some patients infected with COVID-19 being rejected for CC.

Finally, the open-source model proposed is generalisable for hospitals worldwide and potentially for other pandemics. Even though each pandemic inherently exhibits clinical variability, pandemics will always significantly draw from a vast range of hospital resources, including CC. While acknowledging that the model was tailored to Addenbrooke’s hospital, any hospital which reallocated theatre staff to CC can make use of the model and its findings. It can aid hospitals to inform a strategic discussion on resource allocation and prioritisation; a hospital’s individual characteristics, such as different triage, treatments, or resource capacity, can be captured by adjusting the input parameters. Finally, this model could benefit tactical purposes by supporting i) decision-making on opening/closing theatres, ii) active management of shared resources, and iii) pandemic preparation in terms of staff training and recruitment.

## Footnotes

1 RN(CC): Registered Nurse - Critical Care; RN(Scrub): Registered Scrub Nurse; RN(Recovery): Registered Recovery Nurse; RN(ODP/AP): Registered Nurse - Operating Department Practitioner/Anaesthetic Practitioner; RN(In-Charge): Registered Nurse-In-Charge

## Declarations

### Data Transparency

The data can be requested by contacting the authors.
